# Supplementary material for: Cutting in-line with iron: ribosomal function and non-oxidative RNA cleavage
Source: Nucleic Acids Res. 2020 Jul 14;48(15):8663–74. doi: 10.1093/nar/gkaa586 (PMC7470983; doi:10.1093/nar/gkaa586)
Supplement: gkaa586_Supplemental_File [file gkaa586_supplemental_file.pdf]

SUPPLEMENTAL MATERIAL

Cutting in-line with iron: ribosomal function and non-oxidative RNA cleavage

Rebecca Guth-Metzler[1,2†], Marcus S. Bray[2,3†§], Moran Frenkel-Pinter[1,2], Suttipong Suttapitugsakul[1], Claudia Montllor-Albalate[1], Jessica C. Bowman[1,2], Ronghu Wu[1,4], Amit R. Reddi[1,4], C. Denise Okafor[5], Jennifer B. Glass[2,4,6*], and Loren Dean Williams[1,2,4*]

[1]School of Chemistry and Biochemistry, Georgia Institute of Technology, Atlanta, GA, USA, 30332
[2]NASA Center for the Origin of Life, Georgia Institute of Technology, Atlanta, GA, USA, 30332
[3]School of Biological Sciences, Georgia Institute of Technology, Atlanta, GA, USA, 30332
[4]Petit Institute of Bioengineering and Bioscience, Georgia Institute of Technology, Atlanta, GA, USA, 30332
[5]Department of Biochemistry and Molecular Biology, The Pennsylvania State University, University Park, PA, 16802
[6]School of Earth and Atmospheric Sciences, Georgia Institute of Technology, Atlanta, GA, USA, 30332

*To whom correspondence should be addressed: Tel: (404) 385-6258; Fax: (404) 894-2295;

Email: Loren.Williams@chemistry.gatech.edu; Email: Jennifer.Glass@eas.gatech.edu;

[†]These authors contributed equally to this work.

[§]Present Address: Department of Biology, San Diego State University, San Diego, CA, 92182

Supplemental Information:

Supplemental Tables S1-S3

Supplemental Figures S1-S7

SUPPLEMENTAL TABLES

Table S1. Proteins differentially expressed in ribosomes purified from aerobic vs anaerobic cells. Information on metal cofactors was taken from the UniProt entries for each protein.

Condition	Protein more abundant in	Protein name	UniProt ID	Fold more abundant	p-value	Metal cofactor
1 mM added Fe^{2+}	Anaerobic vs. Aerobic	L-threonine dehydratase catabolic TdcB	P0AGF6	211.8	0.02	N.A.
		Hydrogenase maturation factor HypB	P0AAN3	12.1	0.03	N.A.
		Glycerol dehydrogenase	P0A9S5	11.4	0.01	Zn^{2+}
		Phosphoribosylaminoimidazole-succinocarboxamide synthase	P0A7D7	6.0	0.02	N.A.
		Aldehyde-alcohol dehydrogenase	P0A9Q7	6.0	0.03	Fe^{2+}
		Uncharacterized protein YjjI	P37342	5.8	0.02	N.A.
		Probable acrylyl-CoA reductase AcuI	P26646	5.5	0.01	N.A.
		Bacterial non-heme ferritin[a]	P0A998	5.1	0.03	N.A.
		Aspartate ammonia-lyase	P0AC38	4.1	0.01	N.A.
		Fumarate reductase flavoprotein subunit	P00363	3.7	0.05	N.A.
		UvrABC system protein B	P0A8F8	3.6	0.01	N.A.
		Formate acetyltransferase 1	P09373	3.2	0.01	N.A.
		UPF0227 protein YcfP	P0A8E1	3.1	0.02	N.A.
		Phosphoenolpyruvate-protein phosphotransferase	P08839	2.6	0.03	Mg^{2+}
		Ubiquinone/menaquinone biosynthesis C-methyltransferase UbiE	P0A887	2.4	0.02	N.A.
		Glycogen phosphorylase	P0AC86	2.2	0.01	N.A.
		Maltodextrin phosphorylase	P00490	2.1	0.04	N.A.
		Thymidine phosphorylase	P07650	2.1	0.01	N.A.
		Protein-export protein SecB	P0AG86	2.0	0.01	N.A.
		Integration host factor subunit beta	P0A6Y1	2.0	0.04	N.A.
		Cold shock-like protein CspE	P0A972	2.0	0.03	N.A.
		Fatty acid metabolism regulator protein	P0A8V6	1.9	0.02	N.A.
	Aerobic vs. Anaerobic	Lipoyl synthase	P60716	2.2	0.04	[4Fe-4S]
		Translation initiation factor IF-3[b]	P0A707	2.2	0.00	N.A.
		Ribosomal protein S12 methylthiotransferase RimO[b]	P0AEI4	2.3	0.02	[4Fe-4S]
		Dihydrolipoyllysine-residue acetyltransferase component of pyruvate dehydrogenase complex	P06959	2.4	0.01	N.A.
		Ribonucleotide monophosphatase NagD	P0AF24	2.4	0.01	Mg^{2+}
		Ribosomal RNA large subunit methyltransferase K/L[b]	P75864	2.4	0.04	N.A.
		2-oxoglutarate dehydrogenase E1 component	P0AFG3	4.7	0.01	N.A.
		Alkyl hydroperoxide reductase C	P0AE08	5.4	0.05	N.A.
		Pyruvate/proton symporter BtsT	P39396	9.1	0.02	N.A.
		Bifunctional protein PutA	P09546	10.7	0.02	N.A.

No added Fe^{2+}	Anaerobic vs. Aerobic	L-threonine dehydratase catabolic TdcB	P0AGF6	137.5	0.00	N.A.
		Uncharacterized protein YfdQ	P76513	38.0	0.02	N.A.
		Hydrogenase-2 large chain	P0ACE0	31.5	0.01	Ni^{2+}
		Fumarate reductase iron-sulfur subunit	P0AC47	17.5	0.01	[2Fe-2S], [3Fe-4S], [4Fe-4S]
		Glycerol dehydrogenase	P0A9S5	16.9	0.01	Zn^{2+}
		Bacterial non-heme ferritin[a]	P0A998	14.9	0.00	N.A.
		Hydrogenase maturation factor HypB	P0AAN3	11.6	0.02	N.A.
		PFL-like enzyme TdcE	P42632	8.6	0.01	N.A.
		Aldehyde-alcohol dehydrogenase	P0A9Q7	7.2	0.01	Fe^{2+}
		Anaerobic ribonucleoside-triphosphate reductase	P28903	5.8	0.04	N.A.
		Aspartate ammonia-lyase	P0AC38	4.5	0.01	N.A.
		Uncharacterized protein YjjI	P37342	4.3	0.03	N.A.
		SCP2 domain-containing protein YhbT	P64599	4.1	0.01	N.A.
		2,3-bisphosphoglycerate-independent phosphoglycerate mutase	P37689	3.9	0.00	Mn^{2+}
		2-octaprenylphenol hydroxylase	P25535	3.7	0.05	N.A.
		Fructose-1,6-bisphosphatase 1 class 2	P0A9C9	3.3	0.03	Mn^{2+}
		Stationary-phase-induced ribosome-associated protein[b]	P68191	3.3	0.00	N.A.
		Evolved beta-galactosidase subunit alpha	P06864	3.2	0.04	N.A.
		Ribonuclease T	P30014	2.8	0.03	Mg^{2+}
		UPF0227 protein YcfP	P0A8E1	2.5	0.03	N.A.
		UPF0313 protein YgiQ	Q46861	2.4	0.01	[4Fe-4S]
		Methionine--tRNA ligase	P00959	2.4	0.02	Zn^{2+}
		Polyphosphate kinase	P0A7B1	2.4	0.03	Mg^{2+}
		RecBCD enzyme subunit RecC	P07648	2.3	0.02	N.A.
		ADP-heptose--LPS heptosyltransferase 2	P37692	2.2	0.03	N.A.
	Aerobic vs. Anaerobic	RNA-binding protein YhbY	P0AGK4	2.2	0.01	N.A.
		Chaperone protein HscA	P0A6Z1	2.3	0.00	N.A.
		Cytochrome bd-I ubiquinol oxidase subunit 2	P0ABK2	2.3	0.03	heme
		5'-methylthioadenosine/S-adenosylhomocysteine nucleosidase	P0AF12	2.3	0.02	N.A.
		Ribosomal silencing factor RsfS[b]	P0AAT6	2.4	0.00	N.A.
		DNA topoisomerase 4 subunit B	P20083	2.7	0.01	Mg^{2+}
		Lipoyl synthase	P60716	3.0	0.01	[4Fe-4S]
		Transcriptional regulatory protein GlrR	P0AFU4	3.2	0.00	N.A.
		Modulator of FtsH protease HflC	P0ABC3	3.5	0.01	N.A.
		Ribosomal RNA small subunit methyltransferase A[b]	P06992	3.7	0.01	N.A.
		Pyruvate/proton symporter BtsT	P39396	5.8	0.04	N.A.
		Enterobactin synthase component B[a]	P0ADI4	8.0	0.01	Mg^{2+}
		Respiratory nitrate reductase 1 alpha chain	P09152	37.3	0.01	[4Fe-4S] Mo-bis-MGD

[a]Protein is involved in bacterial iron homeostasis; [b]Protein is involved in translation

Table S2. Proteins differentially expressed in ribosomes purified from cells grown with or without 1 mM added Fe^{2+}.
Information on metal cofactors was taken from the UniProt entries for each protein.

Condition	Protein more abundant in	Protein name	UniProt ID	Fold more abundant	p-value	Metal cofactor
Anaerobic	1 mM added Fe vs. without added Fe	Transcriptional regulatory protein OmpR	P0AA16	2.0	0.01	N.A.
		UvrABC system protein B	P0A8F8	2.1	0.03	N.A.
		5'-methylthioadenosine/S-adenosylhomocysteine nucleosidase	P0AF12	2.3	0.04	N.A.
		Large ribosomal RNA subunit accumulation protein YceD[b]	P0AB28	2.3	0.01	N.A.
		ATP-dependent dethiobiotin synthetase BioD 2	P0A6E9	2.7	0.04	Mg^{2+}
		Phosphoribosylaminoimidazole-succinocarboxamide synthase	P0A7D7	2.7	0.01	N.A.
		Phosphoserine phosphatase	P0AGB0	4.1	0.02	Mg^{2+}
	Without added Fe vs. 1 mM added Fe	4-alpha-glucanotransferase	P15977	2.2	0.03	N.A.
Aerobic	1 mM added Fe vs. without added Fe	Probable 4-deoxy-4-formamido-L-arabinose-phosphoundecaprenol deformylase ArnD	P76472	2.0	0.01	N.A.
		GDP-mannose pyrophosphatase NudK	P37128	2.0	0.03	Mg^{2+}
		Uncharacterized protein YfdQ	P76513	2.1	0.02	N.A.
		Uncharacterized protein YciO	P0AFR4	2.1	0.00	N.A.
		ABC transporter ATP-binding protein ModF	P31060	3.1	0.04	N.A.
	Without added Fe vs. 1 mM added Fe	Enterobactin synthase component B[a]	P0ADI4	8.9	0.03	Mg^{2+}
		Fe(3+) dicitrate-binding periplasmic protein[a]	P15028	7.7	0.02	N.A.

[a]Protein is involved in bacterial iron homeostasis
[b]Protein is involved in translation

Table S3: Reaction kinetics of in-line cleavage

	Conditions	k_{obs} (10^{-5} sec^{-1})	No. cleavable phosphates	$[M^{2+}]$ (M)	k/phosphate (10^{-5} sec^{-1}, no. $phosphates^{-1}$, $[M^{2+}]^{-1}$)
This study					
Mg^{2+}, 23S rRNA	pH 7.6, 37°C	5	2905	0.025	0.07
Mg^{2+}, 16S rRNA	pH 7.6, 37°C	3	1541	0.025	0.08
Fe^{2+}, 23S rRNA	pH 7.6, 37°C	67	2905	0.001	23
Fe^{2+}, 16S rRNA	pH 7.6, 37°C	25	1541	0.001	16
Outside studies					
[a]Mg^{2+}, UpU	pH 5.6, 90°C	0.01	1	0.005	2.1
[b]Mg^{2+}, *S(ApG)	pH 9.5, 37°C	0.18	1	0.005	37
[c]Mg^{2+}, polyU	pH 5.6, 90°C	0.32	300	0.005	0.21
[d]Zn^{2+}, ApA	pH 5.1, 90°C	0.64	1	0.01	64
[e]Zn^{2+}, ApA	pH 7.0, 62.1°C	0.06	1	0.001	56
[a]Zn^{2+}, UpU	pH 5.6, 90°C	0.43	1	0.005	85
[f]Zn^{2+}, UpU	pH 7, 80°C	0.04	1	0.001	41
[c]Zn^{2+}, polyU	pH 5.6, 90°C	0.43	300	0.005	0.28
[g]Zn^{2+}, $Up(Tp)_7Tp$	pH 5.5, 37°C	0.50	1	0.01	50

[a]Kuusela & Lönnberg, 1993; [b]Li & Breaker, 1999; [c]Kuusela & Lönnberg, 1994; [d]Kuusela & Lönnberg, 1996; [e]Ikenaga & Inoue, 1974; [f]Breslow & Huang, 1991, [g]Kuusela et al., 1995.

*22 nucleotide DNA-RNA hybrid

SUPPLEMENTAL FIGURES

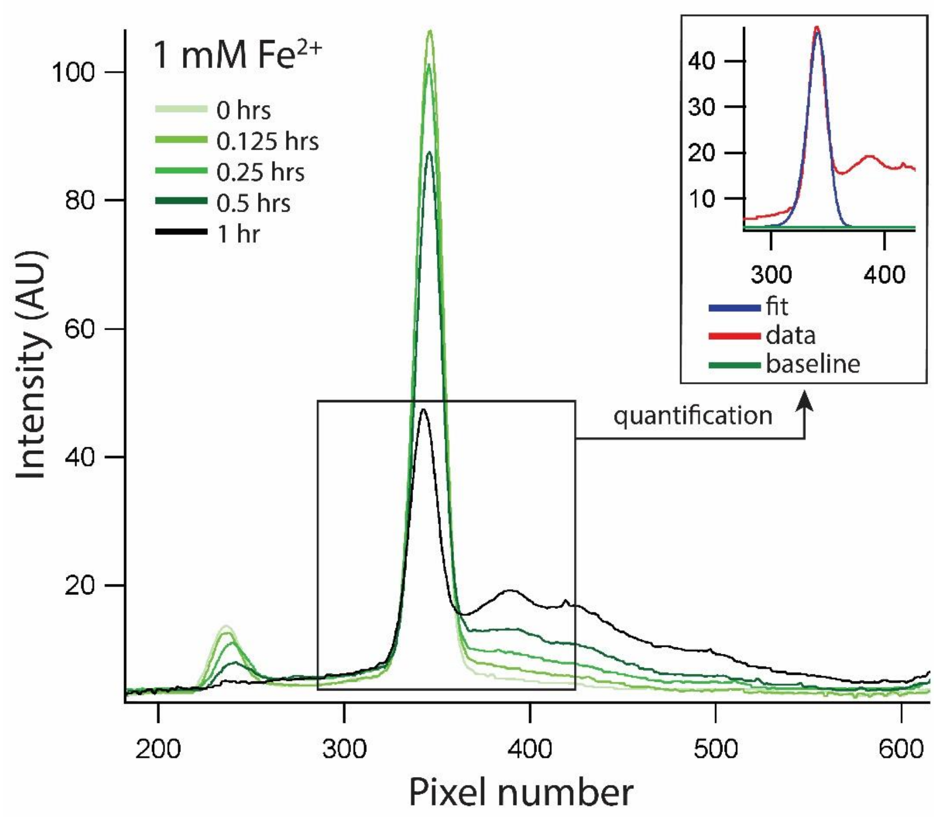

Figure S1. Example quantification of a gel scan for rRNA reactions with 1 mM Fe^{2+}. Shown here is a typical example of lane profiles and peak quantification for rRNA in-line cleavage reactions from a gel scan. Peak fitting was done in Igor Pro 7. The areas of quantified peaks were compiled and plotted in Figure 1.

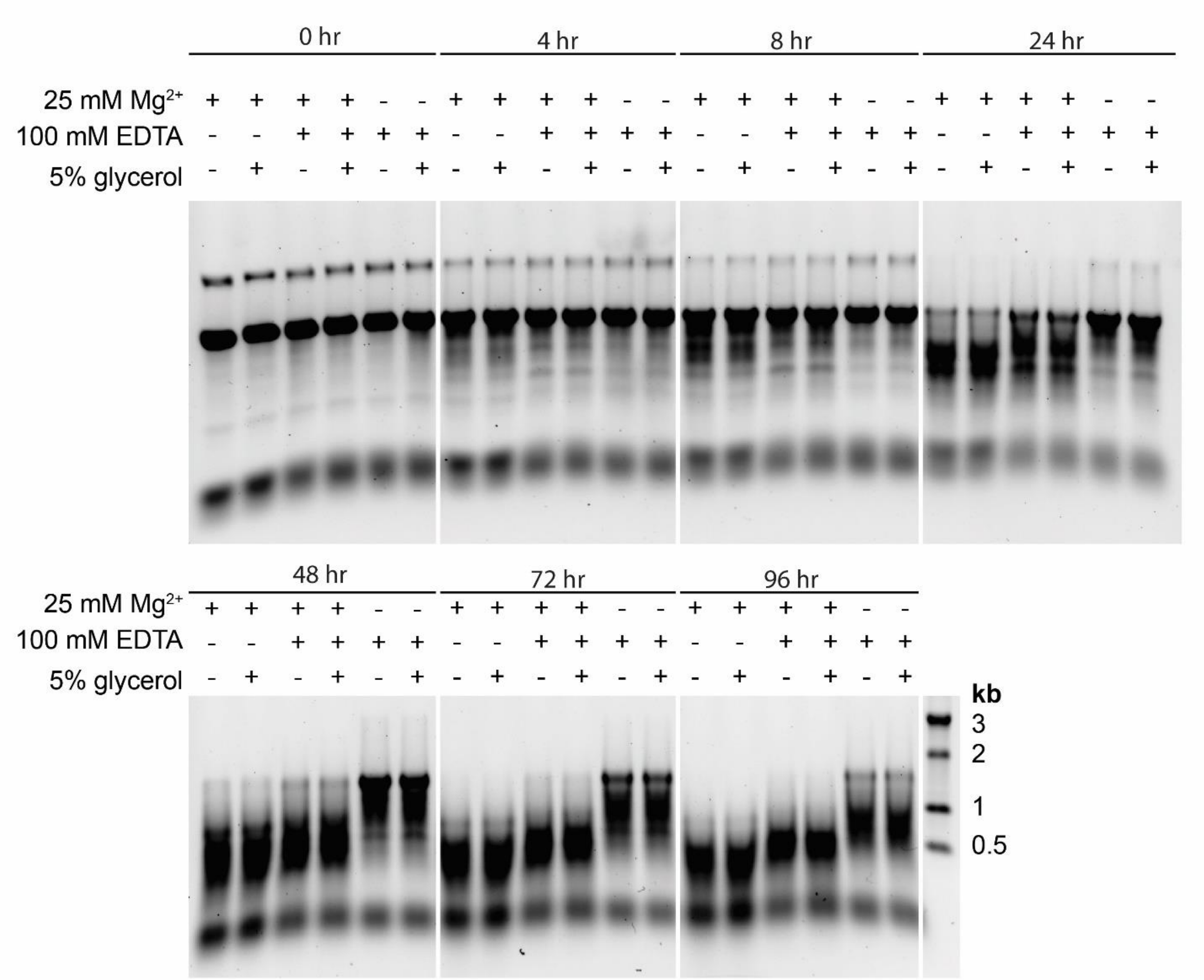

Figure S2. 1% agarose gel showing 5% v/v glycerol does not inhibit Mg^{2+} in-line cleavage of naked rRNA at 37ºC in air over the course of 96 hours. Chelation by 100 mM EDTA inhibits in-line cleavage, but there is no difference with and without 5% glycerol.

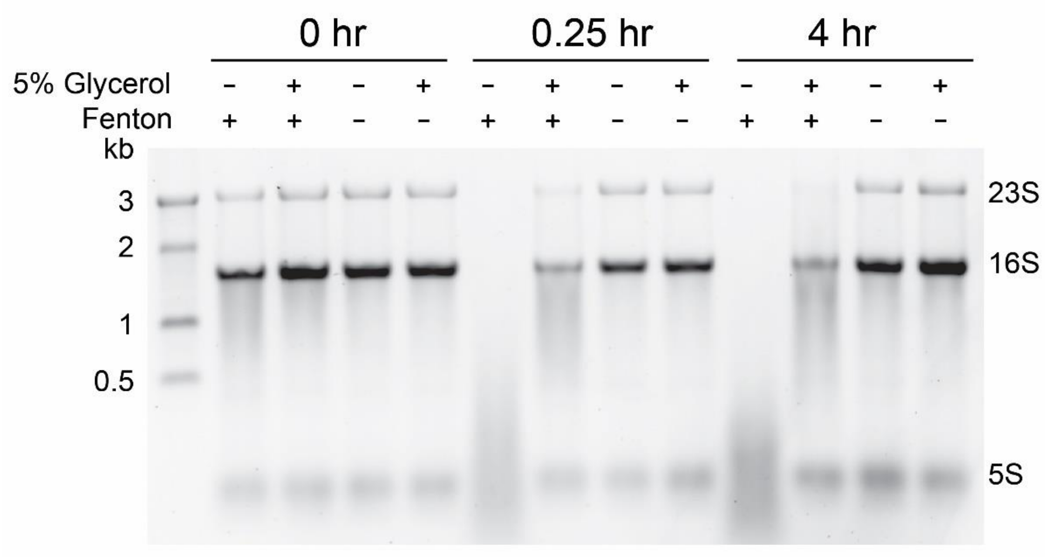

Figure S3. 1% agarose gel showing 5% v/v glycerol inhibition of Fenton chemistry against naked rRNA at 37ºC in air over the course of 4 hours. "+ Fenton" reactions contained 1 mM Fe^{2+}, 0.3% H_2O_2, 10 mM ascorbic acid, and 10 mM EDTA. "- Fenton" reactions contained 10 mM EDTA.

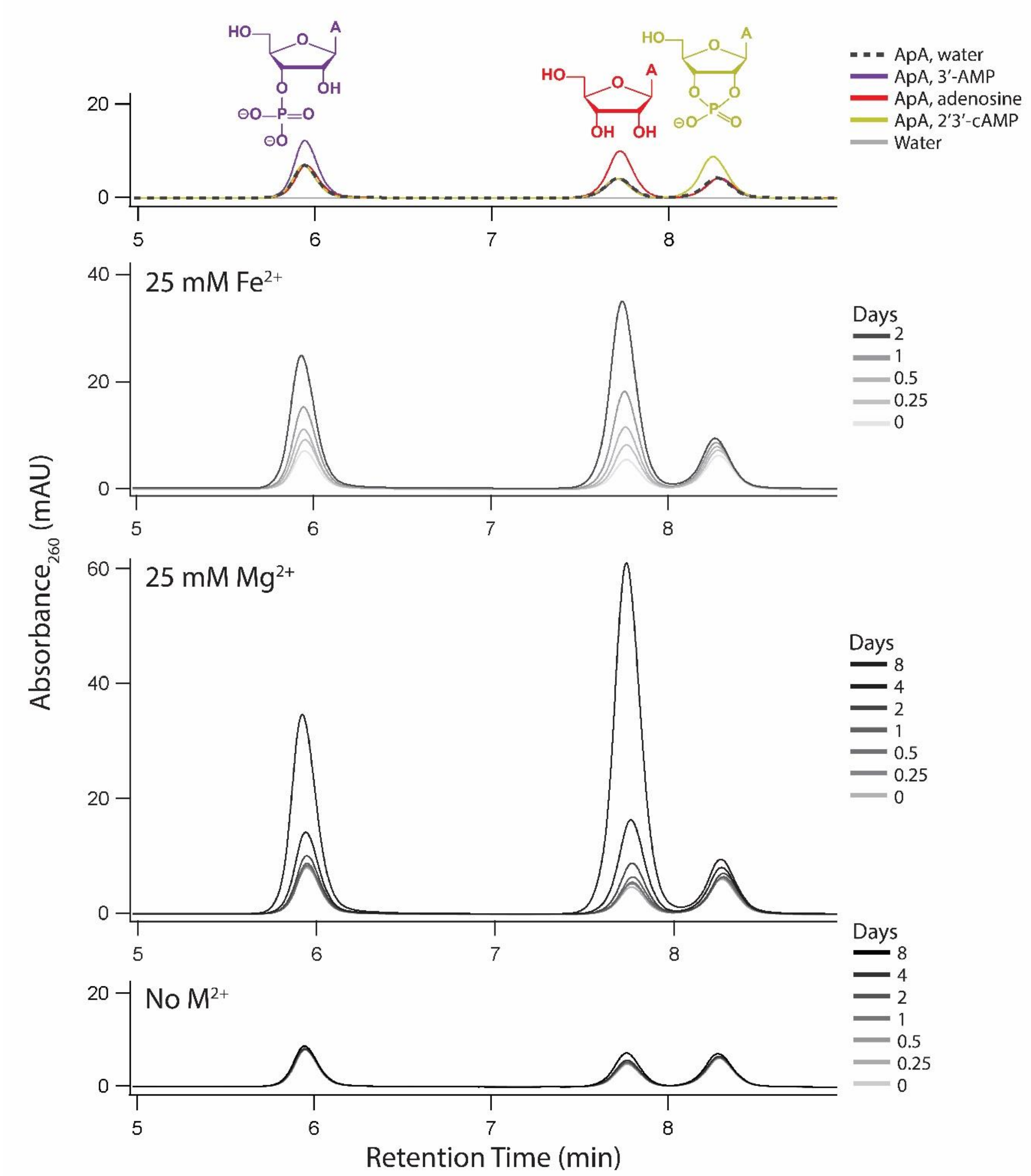

Figure S4. C18 column HPLC chromatograms of ApA reactions showing accumulation of the same products with Fe^{2+} and Mg^{2+}. 3'-AMP, adenosine, and 2',3'-cAMP standards (2.5 µM) were spiked into ApA solutions (0.5 mM ApA, 20 mM HEPES pH 7.6, 30 mM NaCl, 5% v/v glycerol) giving characteristic retention times for 3'-AMP, adenosine, and 2',3'-cAMP. ApA solutions (0.5 mM ApA, 20 mM HEPES pH 7.6, 30 mM NaCl, 5% v/v glycerol) anoxically incubated at 37°C with either 25 mM Fe^{2+} out to 2 days or 25 mM Mg^{2+} out to 8 days accumulate 3'-AMP, adenosine, and 2',3'-cAMP. 2',3'-cAMP peaks build to a lesser extent due to cyclic phosphate hydrolysis to form 3'-AMP or 2'-AMP. There is little cleavage product formation in the no metal (M^{2+}) control.

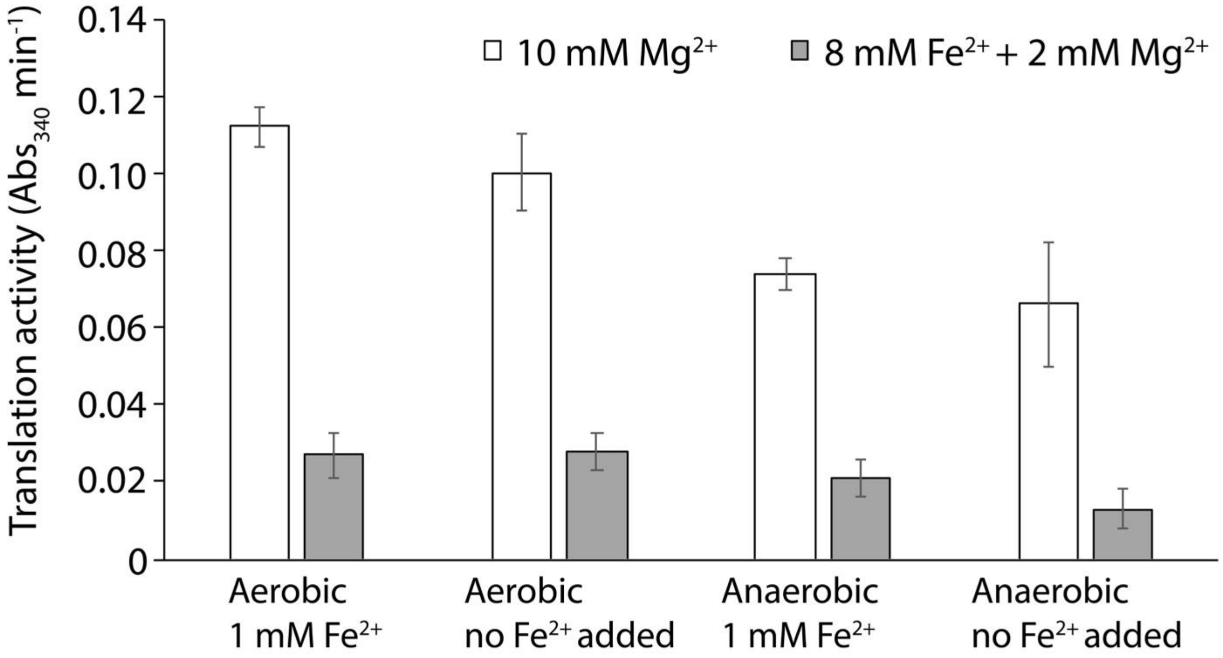

Figure S5. *In vitro* translation activity of purified ribosomes. Production of the protein dihydrofolate reductase (DHFR) from its mRNA was used to monitor translational activity. Protein synthesis was assayed by measuring the rate of NADPH oxidation at Abs_{340} by DHFR. Average values are reported ± standard error of the mean (n=4). All ribosomes were normalized to 9 mg mL^{-1} before adding to translation reactions.

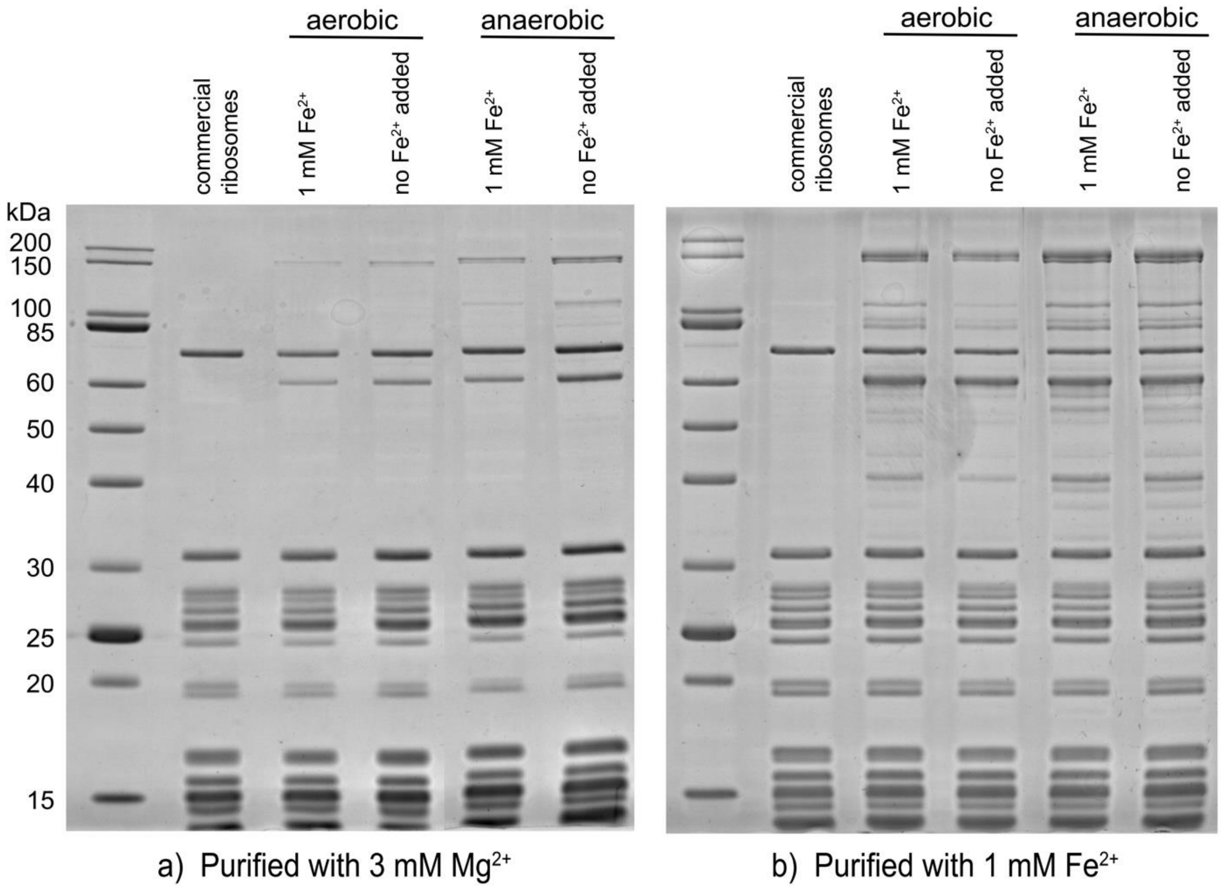

Figure S6. 12% SDS polyacrylamide gels for proteins from ribosomes purified in (a) 3 mM Mg^{2+} or (b) 1 mM Fe^{2+} compared to commercial ribosomes supplied by New England Biolabs.

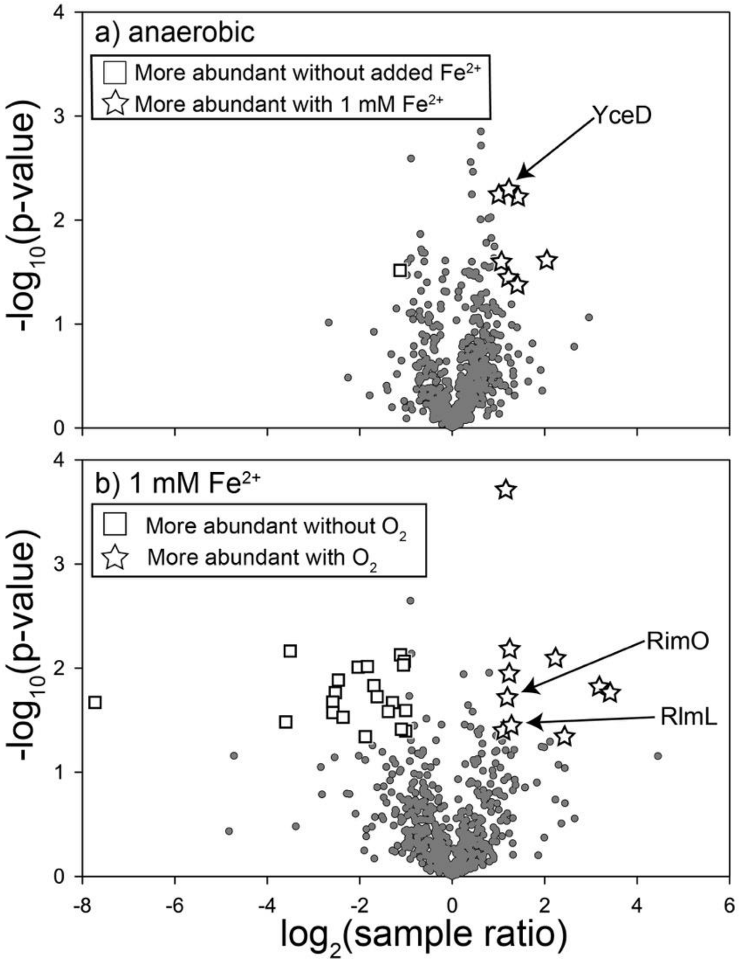

Figure S7. Differential protein abundance between ribosomes purified from cells grown under four growth conditions. Graphs display relative protein abundance in ribosome samples between two growth conditions. Black circles represent proteins not significantly more abundant in either sample. Gray rectangle and white stars represent proteins significantly more abundant in one of the samples. Proteins with a 2-fold or greater abundance in one sample versus another and a p-value less than or equal to 0.05 (n=3), were classified as significantly more abundant.
